# Comprehensive proteomics investigation of *P. vivax*-infected human plasma and parasite isolates

**DOI:** 10.1186/s12879-020-4885-3

**Published:** 2020-03-02

**Authors:** Apoorva Venkatesh, Shalini Aggarwal, Swati Kumar, Srushti Rajyaguru, Vipin Kumar, Sheetal Bankar, Jayanthi Shastri, Swati Patankar, Sanjeeva Srivastava

**Affiliations:** 1 Department of Biosciences and Bioengineering, Indian Institute of Technology Bombay, Powai, Mumbai, India; 2 Department of Microbiology, T. N. Medical College and BYL Nair Hospital, Mumbai, India

**Keywords:** *P. vivax*, Mass spectrometry, Plasma, Biomarkers, Diagnosis, Parasite biology

## Abstract

**Background:**

In recent times, *Plasmodium vivax (P. vivax)* has become a serious threat to public health due to its ability to cause severe infection with fatal outcomes. Its unique biology makes it resilient to control measures that are otherwise effective against *P. falciparum*. A deeper understanding of *P. vivax* biology and pathogenesis is, therefore, essential for developing the right control strategies. Proteomics of *P. falciparum* has been helpful in studying disease biology and elucidating molecular mechanisms involved in the development of disease. However, unlike *P. falciparum*, proteomics data for *P. vivax* infection is minimal due to the absence of a continuous culture system. The dependence on clinical samples and animal models has drastically limited *P. vivax* research, creating critical knowledge gaps in our understanding of the disease. This study describes an in-depth proteomics analysis of *P. vivax*-infected human plasma and parasite isolates, to understand parasite biology, pathogenesis, and to identify new diagnostic targets for *P. vivax* malaria.

**Methods:**

A mass-spectrometry- (MS) based proteomics approach (Q Exactive) was applied to analyze human plasma and parasite isolates from vivax malaria patients visiting a primary health centre in India. Additionally, a targeted proteomics assay was standardized for validating unique peptides of most recurring parasite proteins.

**Results:**

Thirty-eight *P. vivax* proteins were detected in human plasma with high confidence. Several glycolytic enzymes were found along with hypothetical, cytoskeletal, ribosomal, and nuclear proteins. Additionally, 103 highly abundant *P. vivax* proteins were detected in parasite isolates. This represents the highest number of parasite proteins to be reported from clinical samples so far. Interestingly, five of these; three *Plasmodium* exported proteins (PVX_003545, PVX_003555 and PVX_121935), a hypothetical protein (PVX_083555) and Pvstp1 (subtelomeric transmembrane protein 1, PVX_094303) were found in both plasma and parasite isolates*.*

**Conclusions:**

A parasite proteomics investigation is essential to understand disease pathobiology and design novel interventions. Control strategies against *P. vivax* also depend on early diagnosis. This work provides deeper insights into the biology of *P. vivax* by identifying proteins expressed by the parasite during its complex life-cycle within the human host. The study also reports antigens that may be explored as diagnostic candidates.

## Background

*P. vivax* malaria is a public health concern in the tropical and sub-tropical regions of the world. Reflecting this, the estimated vivax malaria burden, in 2017, was around 7.5 million cases worldwide [1] with more people living at risk. The last few years have witnessed a surge in the number of severe vivax malaria cases [2, 3], posing a serious threat to health and economy. The species has unique biological features that support its survival in different climates and geographical regions. An important characteristic is the appearance of gametocytes before the onset of illness which allows transmission even before patients receive treatment [4]. Secondly, the presence of quiescent liver stage forms called hypnozoites trigger multiple episodes of relapse infection from a single infectious mosquito bite [5, 6]. Lower parasite densities and greater vectorial capacity are other features of *P. vivax* that make it different from *P. falciparum*. Therefore, it is important to design and implement interventions specifically targeting *P. vivax* for its control and elimination [7].

Proteomics offers a major advantage in the discovery of new target biomolecules because of its ability to simultaneously characterize proteomes and sub-proteomes [8] without any prior knowledge of the nature of proteins. It also offers great promise in studying protein expressions, protein interactions and protein modifications [9]**.** Mass-spectrometry (MS) qualifies as an excellent tool for the identification and quantification of differentially regulated proteins that provide information about altered pathways and deeper insights into disease biology. Global and stage-specific mass spectrometric analyses of *P. falciparum* in culture have revealed important features of the parasite’s biology [10] including sexual stages [11]. In 2012, Ray and coworkers applied a quantitative proteomics approach to identify differentially expressed host serum proteins in vivax and falciparum malaria patients. The same group also identified host markers of severe infection [12, 13]. Around the same time, Bachmann et al., reported potential muscle damage and microvasculature lesions during the course of cerebral malaria based on elevated muscle protein levels in the plasma of children with cerebral malaria [14]. Recently, an MS-based proteomics study comparing 9 complicated malaria (CM) and 10 uncomplicated malaria (UM) patient samples reported the association of selected *P. falciparum* proteins with the pathophysiology of cerebral malaria [15].

Unlike *P. falciparum,* scientific progress towards understanding *P. vivax* has been minimal, partly due to the inability to maintain continuous cultures for its propagation. Although efforts in this direction have been promising [16] a standard in vitro culture for *P. vivax* has not yet been established. Hence, scientists have had to rely on clinical samples and animal models [17–21] for the study of *P. vivax,* further delaying the identification of new molecules and their functional characterization. Very recently, the proteomes of plasma-derived exosomes from *P. vivax*-infected FRG huHep mice were analyzed to study liver-stage expressed markers of infection. The study revealed human arginase-I and an uncharacterized *P. vivax* protein as potential markers for hypnozoite infection [22]. Proteomics technologies have also facilitated the identification of diagnostic and therapeutic targets. A recent study, investigating plasma samples from falciparum malaria patients, reported four parasite-specific enzymes namely, pHPRT (PF10_0121, parasite hypoxanthine phosphoribosyltransferase,), pPGM (PF11_0208, parasite phosphoglycerate mutase), pLDH (PF13_0141, lactate dehydrogenase) and pFBPA (PF14_0425, fructose bisphosphate aldolase) as possible diagnostic biomarkers [23]. On the contrary, there are no published reports for *P. vivax* malaria, despite a pressing need for new *P. vivax* diagnostic antigens.

Here, we describe a comprehensive proteomics investigation of *P. vivax*, exploring both human plasma and clinical parasite isolates from whole blood. We aim to identify secretory parasite proteins, proteins released upon erythrocyte rupture and abundant proteins expressed by the parasite during their blood-stages within the human host. Our findings provide deeper insights into parasite biology and expand our knowledge of the *P. vivax* clinical proteome. This work also reveals parasite proteins with diagnostic potential and represents an important step in the development of *P. vivax* detection tools. A method for performing validation experiments using targeted proteomics assays is also described.

## Methods

### Sample collection

**Fig. 1:**
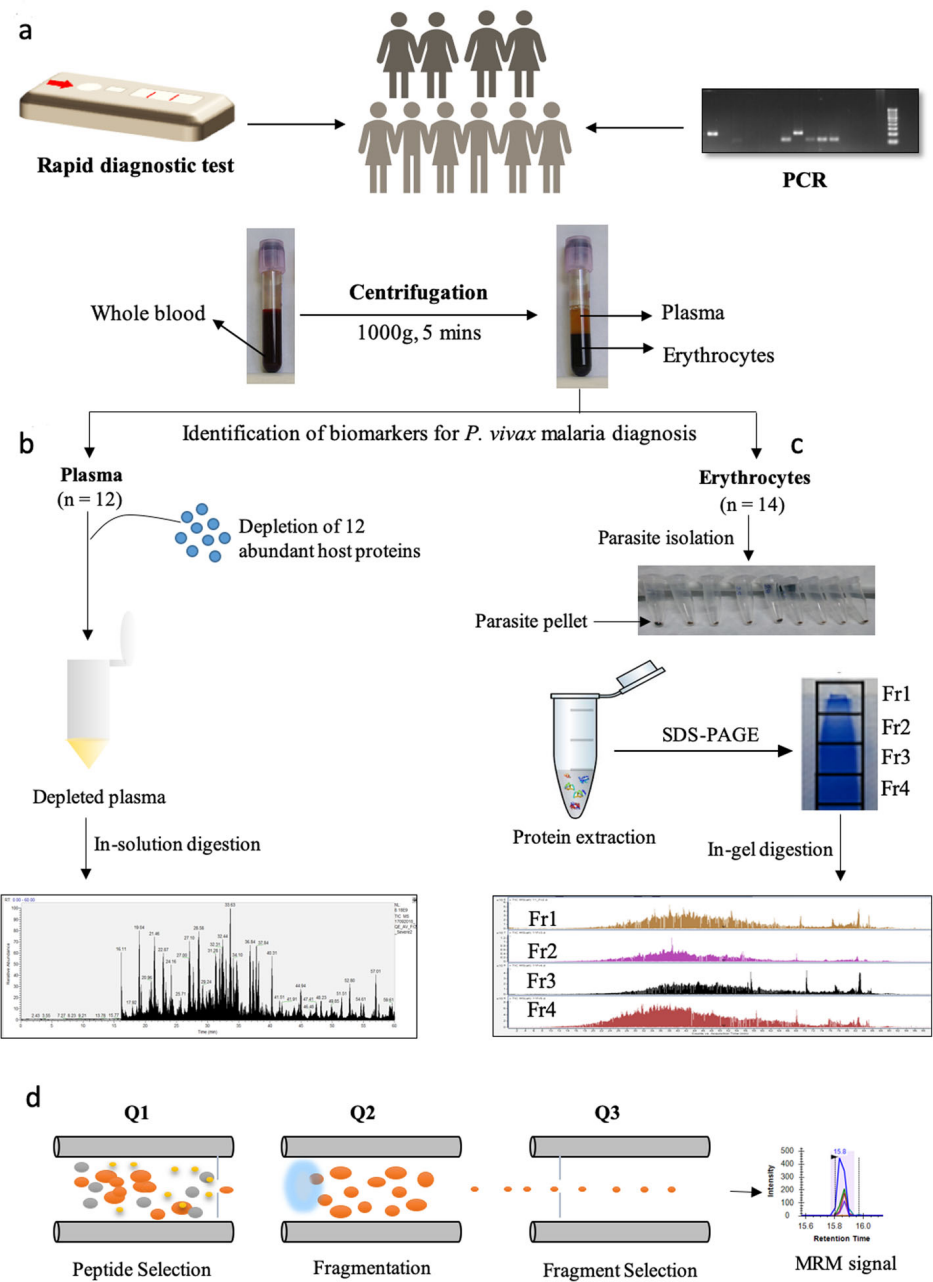
Schematic of the proteomics workflow used for the identification of *P. vivax* parasite proteins in clinical samples. **a** Whole blood samples were confirmed by RDT and PCR for single infection with *P. vivax.* Samples were processed by centrifugation to obtain plasma and erythrocyte fractions. **b** Plasma samples were depleted, quantified, digested and subjected to MS analysis **c** Erythrocyte fractions were saponin-lysed to isolate parasites from infected RBCs (iRBCs). Protein extraction was carried out using parasite lysis buffer, lysates were fractionated using SDS-PAGE and fractions (4–6 fractions) were digested prior to MS analysis. **d** A targeted proteomics approach (MRM assays) was standardized to validate unique peptides of most recurring parasite proteins

Blood samples (2 mL) from 26 patients diagnosed with non-severe vivax malaria (VM) at T. N. Medical College and BYL Nair Hospital, Mumbai were used for the study. These samples represent a small subset of the total number collected from the hospital over 2 years (*n* = 215). Diagnosis was performed using rapid diagnostic tests (FalciVax, Zephyr Biomedicals) and confirmed by nested PCR (Polymerase Chain Reaction) as described previously [20] (Fig. 1a). Written informed consent was taken from all the participants prior to sample collection. For all 26 samples, a few drops (20 μL) of blood were spotted on filter paper disks (GE Healthcare, 1003–090) for PCR before sample processing. Patients detected with mixed infections by PCR were excluded from the study. Plasma samples (*n* = 12) obtained after centrifugation of whole blood were stored at − 80 °C until further use (Fig. 1a). In the second year, parasites were extracted from red blood cell (RBC) pellets using 0.02% saponin lysis mentioned previously [20]. The pellets (*n* = 14) were snap frozen in liquid nitrogen and stored at − 80 °C.

### Study design and sample preparation

Plasma samples from 12 VM patients (VM1 to VM12) were depleted using Spin columns (Pierce Top 12 Thermo Depletion Column, 85,164) to eliminate top 12 abundant proteins (Fig. 1b). Depleted plasma samples were dried and subsequently quantified using Bradford assay. Samples (50 μg) were then denatured using 6 M urea, reduced with TCEP (tris (2-carboxyethyl) phosphine) at 37 °C for 1 h and alkylated using 50 mM IAA (iodoacetamide) for 30 mins in the dark. The concentration of urea was reduced to less than 1 M by addition of 50 mM ABC (Ammonium bicarbonate). Proteins were enzymatically digested into peptides using Trypsin (Promega) by incubating the samples on a thermoshaker for 16 h, at 37 °C. The resulting peptide mixtures were desalted using C-18 columns (3 M EMPORE™) prior to MS analysis.

Parasite pellets were processed as previously described [20]. Briefly, proteins were extracted using parasite lysis buffer (PLB - 4% SDS, 0.5% Triton X-100, 1X PBS and double distilled water). The supernatant fractions were boiled in SDS (Sodium dodecyl sulfate) loading buffer, prior to DTT (dithiothreitol) addition, and then separated by SDS-PAGE (SDS-Polyacrylamide Gel Electrophoresis) (Fig. 1c). Protein lanes were split into 4,6 and 8 fractions depending on the protein profiles and subjected to alternate cycles of rehydration and dehydration using 50 mM ABC and Solution A (Acetonitrile and 50 mM ABC in a 2:1 ratio). Reduction and alkylation were carried out using 10 mM DTT and 10 mM IAA respectively, followed by few more cycles of rehydration and dehydration. The gel pieces were then trypsin digested (1:30) by incubation for 16 h at 37 °C and desalted as described previously.

### MS/MS and data analysis

Digested and desalted peptides (1 μg) from plasma samples were subjected to Data Dependent Nano LC-MS/MS (Liquid chromatography-tandem mass spectrometry) on a Q Exactive mass spectrometer (Thermo Fisher, USA), maintaining a 240 mins gradient time for all the samples. For peptide mixtures obtained from in-gel digested samples (parasite pellets), a Data Dependent Acquisition (DDA) method with a 60 mins gradient time for each fraction was applied on the same instrument mentioned above. Peptide mixtures were injected into Nano-LC at a flow rate of 3 μl/min. Nanospray ESI (Electron Spray Ionization) was used as the ionization source. Eluted peptides from the column (50 cm column –PepMap) were scanned from 350 to 1700 m/z and MS2 (tandem mass spectrometry) from 200 to 2000 m/z at a resolution of 70,000 and 17,500 respectively.

Data files were processed using Trans Proteomic Pipeline (TPP) Software. Raw data files were converted to .mzML format and analyzed using the Comet search engine available within the TPP software. Spectra were searched against *P. vivax* (PlasmoDB) and human (Uniprot) protein databases combined along with decoy and contaminant databases. Stringent statistical identification criteria of 1% protein FDR (false discovery rates) was applied.

### Bioinformatics

*P. vivax* protein sequences were compared against all protein databases available in NCBI (National Center for Biotechnology Information) using pBLAST (protein-basic local alignment search tool). The top *P. falciparum* hits were chosen for each protein and sequence alignment was performed using Clustal Omega. Data for gene ontology and functional categories was obtained from PlasmoDB.

### Optimization of multiple reaction monitoring (MRM) assays for validating protein targets

MRM assays were standardized for peptide validation of important protein targets (Fig. 1d). Peptide lists for 3 target proteins were prepared using DDA data obtained from discovery phase proteomics experiments (Q Exactive, ThermoScientific, USA) described previously. Peptides common among all the technical replicates for that particular protein and unique to the protein were selected. Skyline (MacCoss Lab Software) was used to generate transitions for the selected peptides. For each protein, at least two peptides with a minimum of six transitions were monitored. The dwell time was set to 23 ms. Triple Quadrupole (QQQ, ThermoScientific, USA) MS coupled with UHPLC (Ultra high-performance liquid chromatography, UltiMate 3000, ThermoScientific, USA) was used for the targeted assay. Q1 resolution was set at 0.7 FWHM (full width at half maximum) and Q3 resolution at 1.2 FWHM. The pressure used for collision gas was 2mTorr. Targeted peptides were separated using Hypersil GoldTM (100 mm × 2.1 mm; 1.9 μm) column. Solvent A (MS grade water + 0.1% Formic acid) and Solvent B (80% Acetonitrile in 0.1% Formic acid) with a flow rate of 0.450 mL/min were used to set the gradient method, as follows: 0.01–15 min. 0 to 40% solvent B; 15–17 min. 95% solvent B; 17–18 min. 95% solvent B; 18–19 min., 95 to 5% solvent B; 19–20 min., 5% solvent B. Further optimization was done on the basis of peptide transitions using Skyline. Cycle time (CT) was further optimized and set to 2 s with dwell time of approximately 23 ms. Initially, preliminary experiments using PRM (parallel reaction monitoring) were carried out for validating the findings obtained from the discovery phase (Additional Fig. 1). However, the results were less than satisfactory due to technical difficulties.

## Results

### Identification of *P. vivax* proteins in human plasma

**Fig. 2:**
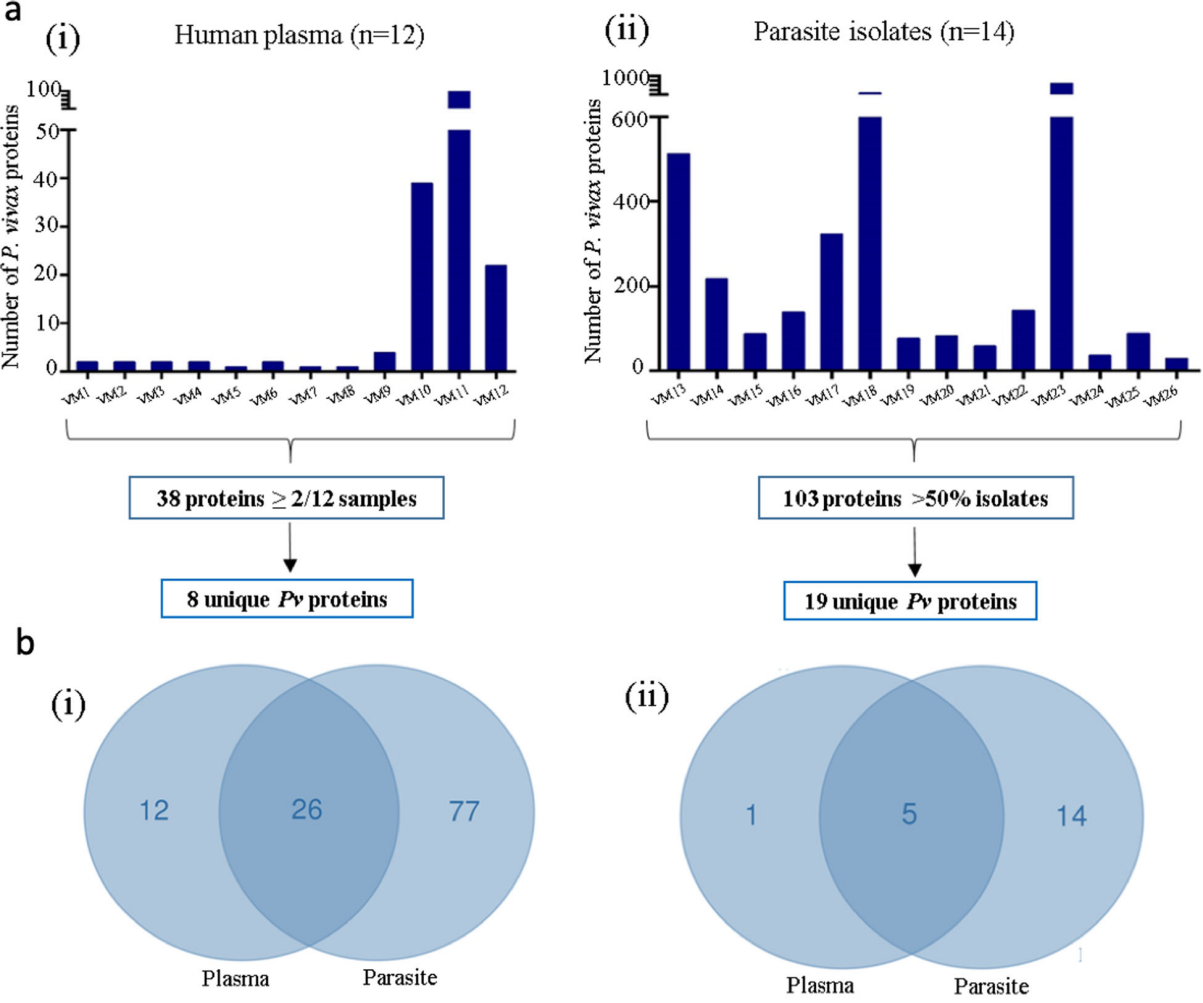
Proteomics analysis of human plasma and parasite clinical isolates from whole blood samples. **a** Number of parasite proteins obtained from (i) 12 plasma samples and (ii) 14 parasite pellets. **b** The Venn diagram displays (i) the total number of *P. vivax* proteins detected in plasma and parasites isolates based on stringent selection criteria (mentioned in Methods section). Of the 38 and 103 proteins in plasma samples and parasite isolates respectively, 26 were found in both sample types. (ii) the total number of *P. vivax-*specific (with no orthologs in *P. falciparum*) proteins detected in plasma (Group B) and parasites isolates

The human plasma proteome was analyzed to identify *P. vivax* proteins that may be secreted during the asexual stages of the parasite’s life cycle and those that persist in circulation after erythrocyte rupture. Based on the total number of parasite proteins detected per individual, samples were segregated into two groups; Group A (1–4 parasite proteins in plasma) and Group B (> 20 parasite proteins in plasma). The majority (75%) of the patients belonged to Group A. The total number of *P. vivax* proteins detected were 2, 2, 2, 2, 1, 2, 1, 1 and 4 for VM1 to VM9 respectively (Fig. 2a.i). As expected, the number of host proteins in plasma were at least hundred times more than the number of *P. vivax* proteins. Group B included 3 patients (VM10, VM11 and VM12) who showed 39, 84 and 22 *P. vivax* proteins in plasma respectively **(**Fig. 2a.i). Strangely, human plasma proteins in these patients were detected in low numbers. Thirty-eight proteins were detected in more than 2 patients (Additional Table 1). A majority were *Plasmodium* exported proteins and heat shock proteins, few were ribosomal proteins and enzymes, while others were proteins involved in DNA binding, transcription and translation. Interestingly, of the eight proteins that were unique to *P. vivax* (Table 1)*,* Pvstp1 (PVX_094303) was detected in 2 patients with 4 peptides each. The other proteins were Pv-fam-d protein (PVX_001650), *Plasmodium* exported proteins (PVX_003545, PVX_003555, PVX_081832 and PVX_121935) and hypothetical proteins (PVX_083555 and PVX_090970).

**Table 1:** List of unique *P. vivax* proteins detected in plasma (A) and parasite isolates (B) by mass spectrometry

S. No	Gene ID	Protein description	Number of samples	Number of peptides
A. Plasma samples				
1	PVX_081832	Plasmodium exported protein	4	1, 1, 1, 1
2	PVX_083555	hypothetical protein	3	1, 4, 2
3	PVX_094303	Pvstp1	2	4, 4
4	PVX_090970	hypothetical protein	2	2, 2
5	PVX_003555	Plasmodium exported protein	2	2, 1
6	PVX_121935	Plasmodium exported protein	2	2, 1
7	PVX_001650	Pv-fam-d protein	2	1, 1
8	PVX_003545	Plasmodium exported protein	2	1, 1
S. No	Gene ID	Protein description	Number of isolates	Number of peptides
B. Parasite Isolates				
1	PVX_094303	Pvstp1, putative	14	6,5,5,2,4,14,3,4,5,12,26,2,2,2
2	PVX_003555	Plasmodium exported protein, unknown function	13	22,15,5,10,4,18,3,4,17,21,2,5,1
3	PVX_003545	Plasmodium exported protein, unknown function	12	3,2,3,1,3,4,2,2,2,7,9,1
4	PVX_092990	tryptophan-rich antigen (Pv-fam-a)	10	15,6,2,5,11,1,1,6,11,1
5	PVX_092995	tryptophan-rich antigen (Pv-fam-a)	10	1,11,2,8,5,2,2,5,15,2
6	PVX_096975	VIR protein	9	1,6,1,4,2,1,3,6,1
7	PVX_112670	unspecified product	9	4,5,2,2,2,2,1,1,4
8	PVX_090265	tryptophan-rich antigen (Pv-fam-a)	9	2,14,3,5,4,3,4,21,3
19	PVX_001685	Phist protein (Pf-fam-b)	8	7,2,3,1,4,3,7,1
10	PVX_094295	Pv-fam-h protein	8	3,1,1,1,2,4,1,1
11	PVX_096995	tryptophan-rich antigen (Pv-fam-a)	8	12,4,4,6,3,13,1,1
12	PVX_118695	Pv-fam-d protein	8	10,6,1,1,10,3,17,1
13	PVX_121935	Plasmodium exported protein, unknown function	8	3,4,4,2,4,2,10,3
14	PVX_083555	hypothetical protein	7	2,1,2,1,5,11,2
15	PVX_090275	tryptophan-rich antigen (Pv-fam-a)	7	1,1,4,4,1,2,16
16	PVX_096985	variable surface protein Vir, putative	7	3,4,1,1,7,1,1
17	PVX_101515	tryptophan-rich antigen (Pv-fam-a)	7	6,1,2,3,4,12,1
18	PVX_112110	unspecified product	7	1,5,4,7,3,1,1
19	PVX_115460	Plasmodium exported protein, unknown function	7	2,1,1,1,2,2,3

The term *unique* indicates the absence of orthologs in *P. falciparum* determined by sequence alignment of *P. vivax* proteins against top *P. falciparum* hits using Clustal Omega

### Proteomic analysis of *P. vivax* isolates reveals abundant parasite proteins

In order to identify proteins that are highly expressed by blood stage parasites, an in-depth proteomic analysis of isolated parasites was performed. Proteins were considered ‘highly expressed’ or ‘abundant’ depending on their frequency of detection across patients and not protein amount in a sample. More than 100 parasite pellets were processed as previously described [20], however data from only 14 pellets are reported in this study due to several biological and technical limitations (Fig. 2a.ii). The total number of parasite proteins largely varied among all the samples. Five hundred and thirteen, 218, 88, 139, 322, 645, 77, 83, 59, 143, 29, 841, 37 and 89 parasite proteins were detected in samples VM13 to VM 26 using mass spectrometry respectively. Hundred and three proteins were detected in more than 50% of the isolates (Fig. 2a.ii) which included many 60S and 40S ribosomal proteins, heat shock proteins, translation initiation factors, enzymes, hypothetical proteins, *Plasmodium* exported proteins, PHIST (*Plasmodium* helical interspersed subtelomeric) proteins and cytoskeletal proteins among others **(**Additional Table 1). Of these, 19 were unique to *P. vivax* (Table 1) showing very low identity with *P. falciparum* proteins (Additional Table 2). Interestingly, Pvstp1 (PVX_094303) was identified in all 14 samples, followed by *Plasmodium* exported proteins (PVX_003555 and PVX_003545) in 13 and 12 patients respectively. These proteins were also detected in few plasma samples along with *Plasmodium* exported protein (PVX_121935) and hypothetical protein (PVX_083555). Several members of the PvTRAg (tryptophan-rich antigen, Pv-fam-a) family were found to be highly expressed by the parasite. Other proteins of significance were VIR (variant interspersed repeats), PHIST proteins and certain unspecified products. In summary, twenty-six parasite proteins were identified in both plasma and parasite isolates (Additional Table 3), of which five of the aforementioned proteins were specific to *P. vivax* (Table 2, Fig. 2b).

**Table 2:** List of unique *P. vivax* proteins detected in both plasma and parasite isolates by mass spectrometry

S. No	Gene ID	Product Description	^a^Literature (PMID)
1	PVX_003545	Plasmodium exported protein, unknown function	18,843,361, 28,841,253, 22,028,927, 21,515,433, 25,545,414
2	PVX_003555	Plasmodium exported protein, unknown function	18,843,361, 25,106,850, 22,028,927, 21,515,433, 25,545,414
3	PVX_083555	hypothetical protein	18,843,361, 28,841,253, 22,028,927, 21,515,433, 25,545,414
4	PVX_094303	Pvstp1, putative	28,841,253
5	PVX_121935	Plasmodium exported protein, unknown function	18,843,361, 21,515,433

^a^Some information on these *P. vivax* proteins can be found in the following articles

### Gene-ontology (GO) classification and bioinformatics provide new insights

Species specificity of important protein candidates was confirmed by sequence alignment with corresponding *P. falciparum* hits obtained after BLAST search (Additional Fig. 2). Interestingly, percentage identity to *P. falciparum* proteins was very low and almost nil when compared to host protein sequences (Additional Table 2). Additional Fig. 2 highlights certain segments of the protein sequences having highest dissimilarity between the two species, confirming that the proteins identified were indeed specific to *P. vivax.* These proteins represent candidates with highest diagnostic potential in terms of their high abundance in parasite isolates, presence in plasma and specificity to *P. vivax*.

**Fig. 3:**
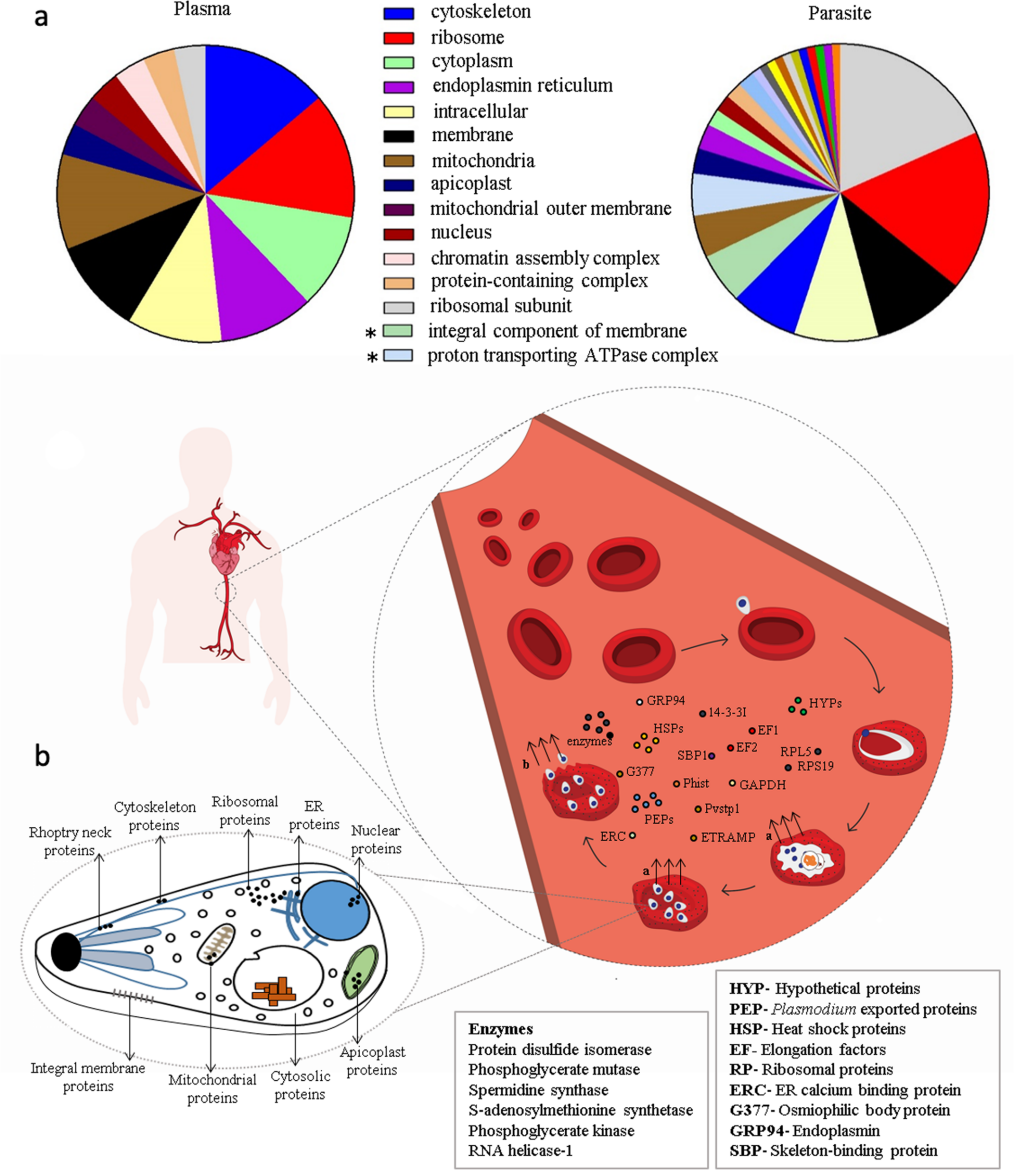
Functional classification of parasite proteins from plasma and parasite isolates **a** Pie chart represents computed GO components for proteins found from plasma and parasite isolates. The categories mentioned in the figure were found in both biological sources except the ones marked with an asterisk * (Present in parasite isolates only). **b** An overview of the parasite proteins identified in plasma and parasite isolates. This figure maps only a few proteins out of the total proteins detected using MS analysis. Individual protein groups are represented based on their location within the parasite

In order to compare the nature of proteins detected in the two clinical sources, 38 and 103 parasite proteins from plasma and parasite isolates respectively were studied. Owing to the unavailability of a culture system for *P. vivax* propagation, GO data were available for only 50% of the total proteins. Many of the proteins in circulation were ribosomal and cytoskeletal proteins (13.7%). Proteins of the cytoplasm and endoplasmic reticulum as well as other membrane, intracellular and mitochondrial proteins represented the next major category of parasite proteins in circulation **(**Fig. 3a, Additional Table 4A). Interestingly, many enzymes were also found. Similar proteins were found to be highly abundant in parasite isolates, however there were few striking differences (Additional Table 4B). Integral membrane proteins were undetected in plasma samples. A few other proteins of the microsomes, mitochondrial matrix, nucleosome, phosphopyruvate hydratase complex, plasma membrane, proteasome complex and symbiont vacuole membrane were also not represented in the plasma proteome. Ribosomal subunit proteins formed the major group of proteins in clinical isolates, with little presence in the plasma. An integrated view of *P. vivax* proteins in clinical samples is shown in Fig. 3b based on proteomics findings. None of these proteins were detected in dengue and healthy samples that were used as controls in the experiment.

### Validation of peptides using MRM assays

Targeted proteomics experiments using MRM assays were performed for 13 peptides (Additional Table 5). Four peptides out of 13 showed consistent results among all the 8 plasma samples in the initial round of experiments, of which two mapped to parasite protein Pvstp1. Peak allocation was carried out based on the most consistent retention time (RT) across multiple runs. Results of the preliminary experiments are shown in Additional Fig. 3. Together these results, for the first time, provide hope for peptide and protein validation with clinical samples using MRM assays in future.

## Discussion

Plasma mirrors the pathophysiological state of any diseased individual [24]. In case of malaria, the study of human plasma is significant because RBCs represent sites of maximum parasite activity. Parasites grow and multiply within RBCs and subsequently rupture them to enter the bloodstream and invade other uninfected RBCs. In the process, several proteins are released into circulation. Identifying the presence of these circulating parasite proteins in plasma is crucial in the context of parasite biology and disease pathogenesis. Some proteins may also be important diagnostic antigens, if they can be easily detected and measured. However, the complex nature of human plasma and presence of several abundant host proteins, mask their detection.

Recent advancements in mass spectrometry provide a sensitive platform for studying complex proteomes, but the dynamic range of plasma poses a serious challenge to MS-based proteomics. So far, only a few thousand human proteins have been discovered by LC-MS/MS, while parasite proteins are completely masked by the abundant human proteins present in concentrations that range from 50 mg/mL to as low as 5 pg/mL [25]. In this study, plasma samples from VM patients were first depleted to eliminate abundant host proteins like serum albumin, apolipoproteins, immunoglobulins, haptoglobin, fibrinogen, macroglobulin, transferrin and a few others. The number of *P. vivax* proteins obtained after MS analysis varied largely among the 12 patients. Nine patients showing only 1 or 2 parasite proteins in their plasma were grouped together (Group A). The other 3 patients (Group B) displayed a ratio distortion with significantly higher parasite proteins accompanied by a surprisingly lower number of human proteins. Unfortunately, these disparate observations could not be correlated to the phenotypes of health and disease as clinical information for these patients was not available. The most important data missing was the parasite density for each sample which could have explained the differences observed between group A and group B samples.

In this study, we integrate the findings from human plasma and parasite proteomes to understand *P. vivax* biology. The data also reveals novel protein targets that may be considered further for evaluation as diagnostic markers for vivax malaria.

Several hypothetical proteins were identified in human plasma among other exported proteins with unknown functions. According to a comparative genome study published few years ago, almost half the genes known to have orthologs in *P. falciparum, P. knowlesi* and *P. yoelii,* encode conserved hypothetical proteins [26]. Therefore, their detection in large numbers was expected. Unfortunately, due to lack of *P. vivax* functional assays*,* hypothetical proteins along with several others remain functionally uncharacterized even today. Heat shock proteins (HSPs, molecular chaperons) constituted the third class of proteins in human plasma. In *P. falciparum* malaria, HSP70 was previously shown to mediate protective immunity [27]. Recently, PvHSP70 was also characterized and evaluated for its serodiagnostic applicability [28]. We speculate that its presence in plasma, as determined from this study, could be one reason for the high seroreactivity observed among malaria positive patients in previous studies. The detection of cytoskeletal, ribosomal, and nuclear proteins majorly reflects parasite lysis during infection as most of these proteins are not secretory in nature. On the contrary, several glycolytic enzymes that were found in circulation may indeed be secreted during different stages of the lifecycle or released upon invasion or erythrocyte rupture. Glycolytic proteins such as PGK (phosphoglycerate kinase), Protein disulfide isomerase and GAPDH (Glyceraldehyde 3-phosphate dehydrogenase), previously reported in the context of malaria [29–31] were detected in plasma with high confidence. Interestingly, a recent study explored the role of GAPDH as a new malaria diagnostic biomarker for *P. falciparum*. The authors reported high antibody levels against epitopes specific for *P. falciparum* [31]. Apart from these proteins, few others like PHIST, ETRAMP (early transcribed membrane protein), ran binding protein 1, Pvstp1, skeleton-binding protein 1 and cytoplasmic and nuclear enzymes were found. More importantly, four *Plasmodium* exported proteins, two hypothetical proteins, Pvstp1 and one protein of the Pv-fam-d family were found to be unique to *P. vivax*. Although these proteins represent good leads, it is important to mention that we were unable to detect many other parasite proteins present at extremely low concentrations, despite using highly sensitive MS technologies.

In order to improve the parasite proteome coverage, alternative methods to overcome the existing challenges in plasma biomarker discovery must be explored. One strategy to greatly enhance the in-depth analysis of plasma proteomes could be the use of fractionation methods and other protein separation techniques such as SDS-PAGE prior to MS analysis, Alternate strategies involve the use of Data independent acquisition (DIA), a superior technique in MS which fragments every single peptide in a sample, unlike DDA. This technique permits an unbiased acquisition of data and provides larger number of peptides with greater reproducibility [32]. Other alternatives to improve plasma proteome coverage for biomarker discovery have been extensively described by Geyer and coworkers in their article [25].

A comprehensive analysis of 14 parasite isolates using advanced Orbitrap technology revealed many novel proteins that have never been identified previously from clinical samples. Very interestingly, ribosomal subunit proteins, integral membrane proteins, proton transporting ATPase complexes formed a major group of highly expressed parasite proteins which were not found in plasma. Of particular interest were several members of the PvTRAg (Pv-fam-a) gene family which could not be identified previously using less sensitive mass spectrometers. Consistent with previous findings, *Plasmodium* exported proteins and hypothetical proteins were found to be highly abundant. Surprisingly, none of the merozoite surface proteins (MSPs), except MSP 8, were detected in both parasite isolates and plasma. Twenty-six parasite proteins were identified in both plasma and parasite isolates, of which 5 were unique to *P. vivax.* Pvstp1 was particularly interesting because it was detected in all 14 parasite isolates as well as in plasma. Further evaluation of these proteins as potential markers for *P. vivax* because of their specificity and high abundance (based on frequency of occurrence in vivax patients) is highly recommended. A preliminary validation of our findings was performed by adopting a targeted-proteomics based approach (MRM assays) for selected peptides. Reliable assays were successfully generated that could confidently identify target peptides in clinical samples. While this opens up new avenues for parasite biomarker validation in clinical samples, we are still far away from understanding the effect of variable criteria on the quality of results. To address the problems associated with the interpretation of Targeted MS assays, it is important to apply strict procedures and guidelines to establish more confidence in the data [33]. To overcome some of these challenges, we intend to include synthetic peptides in our assays, using concentrated samples in future.

## Conclusion

Proteome studies are expected to contribute significantly to malaria research, by facilitating the detection of novel protein targets and pathways involved in malaria pathophysiology. Using advanced MS technologies, our study reveals several convincing candidates, found to be abundant in plasma as well as in clinical parasite isolates. This work provides new insights into *P. vivax* biology and hope in the area of vivax malaria diagnosis. It represents an important resource for further follow-up studies using larger clinical cohorts.

## Supplementary information

**Additional file 1: Table 1.** *P. vivax* proteins in plasma samples and parasite isolates. **Table 2.** Sequence alignment data for unique proteins obtained from parasite pellets using pBLAST. **Table 3.** *P. vivax* proteins found in both plasma and parasite isolates. **Table 4A.** GO classification of *P. vivax* proteins detected in plasma. **Table 4B.** GO classification of *P. vivax* proteins detected in parasite isolates. **Table 5.** List of proteins and peptides selected for the MRM assay. **Figure 1.** Representative peptides unique to *P. vivax* monitored using PRM assay. **Figure 2.** Sequence alignment of unique *P. vivax* proteins. **Figure 3.** Standardization of peptide validation experiments using a targeted proteomics approach.

## Data Availability

The datasets generated during and/or analysed during the current study are available in PRIDE, with ID - PXD015165.

## Abbreviations

ABC: Ammonium bicarbonate
cm: centimeter
CM: Complicated malaria
CT: Cycle time
DDA: Data Dependent Acquisition
DIA: Data Independent Acquisition
DNA: Deoxyribose nucleic acid
DTT: Dithiothreitol
ESI: Electron Spray Ionization
ETRAMP: Early transcribed membrane protein
FDR: False Discovery Rate
FRG: Fah, Rag, and Ilrg genes knockked out model
FWHM: Full width at half maximum
GAPDH: Glyceraldehyde 3-phosphate dehydrogenase GAPDH
GO: Gene-ontology
HSP: Heat Shock Protein
IAA: Iodoacetamide
iRBCs: Infected RBCs
LC-MS: Liquid chromatography- Mass spectrometry
M: Molar
min: Minute
mM: Millimolar
MRM: Multiple Reaction Monitoring
ms: Milliseconds
MS2 and MS/MS: Tandem mass spectrometry
NCBI: National Center for Biotechnology Information
pBLAST: Protein-basic local alignment search tool
PBS: Phosphate buffered saline
PCR: Polymerase Chain Reaction
pFBPA: Parasite Fructose bisphosphate aldolase
PHIST: Plasmodium helical interspersed subtelomeric
pHPRT: Parasite Hypoxanthine phosphoribosyltransferase
PLB: Parasite Lysis Buffer
pLDH: Parasite Lactate dehydrogenase
pPGM: Parasite Phosphoglycerate mutase
PRM: Parallel Reaction Monitoring
Pvstp: *Plasmodium vivax* subtelomeric transmembrane protein
PvTRAg: *Plasmodium vivax* tryptophan-rich antigen
QQQ: Triple Quadrupole
RT: Retention Time
s: Seconds
SDS: Sodium dodecyl sulfate
SDS-PAGE: SDS Polyacrylamide Gel Electrophoresis
TCEP: Tris (2-carboxyethyl) phosphine
TPP: Trans Proteomic Pipeline
UHPLC: Ultra High-Performance Liquid Chromatography
UM: Uncomplicated malaria
VIR: Variant interspersed repeats
VM: Vivax Malaria
