# Supplementary material for: Comprehensive proteomics investigation of P. vivax-infected human plasma and parasite isolates
Source: BMC Infect Dis. 2020 Mar 2;20:188. doi: 10.1186/s12879-020-4885-3 (PMC7053139; doi:10.1186/s12879-020-4885-3)

Additional Information

Additional table 1. *P. vivax* proteins in plasma samples and parasite isolates (Criteria: Number of patients, number of peptides, 1% FDR cut-off)

PLASMA SAMPLES			
Proteins found in ≥ 2/12 plasma samples			
**S.No**	**Accession ID**	**Protein description**	**Number of patients**
1	PVX_099315	heat shock protein 70	4
2	PVX_081832	Plasmodium exported protein	4
3	PVX_083555	hypothetical protein location	3
4	PVX_083560	Plasmodium exported protein	3
5	PVX_087950	heat shock protein 86	3
6	PVX_089425	heat shock 70 kDa protein	3
7	PVX_091515	GTP-binding nuclear protein RAN/TC4	3
8	PVX_093680	Phist protein (Pf-fam-b) location	3
9	PVX_094635	tubulin beta chain	3
10	PVX_097583	skeleton-binding protein 1	3
11	PVX_099525	S-adenosylmethionine synthetase	3
12	PVX_099535	phosphoglycerate kinase	3
13	PVX_101200	actin location	3
14	PVX_115450	hypothetical protein	3
15	PVX_117322	glyceraldehyde-3-phosphate dehydrogenase	3
16	PVX_001650	Pv-fam-d protein location	2
17	PVX_003545	Plasmodium exported protein	2
18	PVX_003555	Plasmodium exported protein	2
19	PVX_081265	chromatin assembly factor 1 protein WD40 domain	2
20	PVX_082845	elongation factor 1-gamma	2
21	PVX_084940	voltage-dependent anion-selective channel protein	2
22	PVX_085275	60S ribosomal protein L5	2
23	PVX_088960	protein disulfide isomerase	2
24	PVX_089505	14-3-3 protein	2
25	PVX_090070	ran binding protein 1	2
26	PVX_090160	40S ribosomal protein S19	2
27	PVX_091105	endoplasmic reticulum-resident calcium binding protein	2
28	PVX_091640	phosphoglycerate mutase	2
29	PVX_092065	spermidine synthase	2
30	PVX_094303	Pvstp1	2
31	PVX_095000	heat shock protein 60	2
32	PVX_096070	early transcribed membrane protein (ETRAMP) location	2
33	PVX_101400	osmiophilic body protein	2
34	PVX_117030	RNA helicase-1	2
35	PVX_117925	elongation factor 2	2
36	PVX_121935	Plasmodium exported protein	2
37	PVX_123745	endoplasmin	2
38	PVX_090970	hypothetical protein	2
**PARASITE ISOLATES**			
Proteins found in ≥ 50 % parasite isolates			
S.No	**Accession ID**	**Product Description**	**Number of patients**
1	PVX_094303	Pvstp1, putative	14
2	PVX_093680	Phist protein (Pf-fam-b)	14

3	PVX_115450	hypothetical protein, conserved	14
4	PVX_003555	Plasmodium exported protein, unknown function	13
5	PVX_087950	heat shock protein 86, putative	13
6	PVX_089425	heat shock 70 kDa protein, putative	13
7	PVX_094635	tubulin beta chain, putative	13
8	PVX_097583	skeleton-binding protein 1, putative	13
9	PVX_101200	actin	13
10	PVX_003545	Plasmodium exported protein, unknown function	12
11	PVX_083560	Plasmodium exported protein, unknown function	12
12	PVX_089505	14-3-3 protein, putative	12
13	PVX_092070	parasitophorous vacuolar protein 1, putative	12
14	PVX_095000	heat shock protein 60, putative	12
15	PVX_099315	heat shock protein 70, putative	12
16	PVX_099525	S-adenosylmethionine synthetase, putative	12
17	PVX_117030	RNA helicase-1, putative	12
18	PVX_121930	Plasmodium exported protein, unknown function	12
19	PVX_080100	multidrug resistance protein 1, putative	11
20	PVX_092065	spermidine synthase, putative	11
21	PVX_118145	40S ribosomal protein S5, putative	11
22	PVX_092990	tryptophan-rich antigen (Pv-fam-a)	10
23	PVX_092995	tryptophan-rich antigen (Pv-fam-a)	10
24	PVX_088960	protein disulfide isomerase, putative	10
25	PVX_091470	heat shock protein 101, putative	10
26	PVX_100835	conserved protein, unknown function	10
27	PVX_101400	osmiophilic body protein, putative	10
28	PVX_123340	eukaryotic translation initiation factor 3 subunit 10, putative	10
29	PVX_096975	VIR protein	9
30	PVX_112670	unspecified product	9
31	PVX_090265	tryptophan-rich antigen (Pv-fam-a)	9
32	PVX_083240	6-cysteine protein	9
33	PVX_083515	protein disulfide-isomerase, putative	9
34	PVX_091515	GTP-binding nuclear protein RAN/TC4, putative	9
35	PVX_091700	circumsporozoite-related antigen, putative	9
36	PVX_095080	40S ribosomal protein S3A, putative	9
37	PVX_096070	early transcribed membrane protein (ETRAMP)	9
38	PVX_097915	60S ribosomal protein L4, putative	9
39	PVX_114315	hexokinase, putative	9
40	PVX_116915	exported protein 2, putative	9
41	PVX_117170	40S ribosomal protein S3, putative	9
42	PVX_117322	glyceraldehyde-3-phosphate dehydrogenase, putative	9
43	PVX_117615	signal peptide peptidase, putative	9
44	PVX_117625	V-type H(+)-translocating pyrophosphatase, putative	9
45	PVX_001685	Phist protein (Pf-fam-b)	8
46	PVX_094295	Pv-fam-h protein	8
47	PVX_096995	tryptophan-rich antigen (Pv-fam-a)	8
48	PVX_118695	Pv-fam-d protein	8
49	PVX_121935	Plasmodium exported protein, unknown function	8
50	PVX_002650	40S ribosomal protein S26, putative	8
51	PVX_003665	hexose transporter	8
52	PVX_003905	6-cysteine protein	8
53	PVX_080440	40S ribosomal protein S11, putative	8

54	PVX_082840	60S ribosomal protein L6, putative	8
55	PVX_082845	elongation factor 1-gamma, putative	8
56	PVX_083045	phosphoethanolamine N-methyltransferase	8
57	PVX_089635	phosphoglucomutase-2, putative	8
58	PVX_090155	tubulin alpha chain, putative	8
59	PVX_094400	60S ribosomal protein L13a, putative	8
60	PVX_094535	RNA-binding protein, putative	8
61	PVX_095015	enolase, putative	8
62	PVX_096265	40S ribosomal protein S5, putative	8
63	PVX_099055	alkaline phosphatase, putative	8
64	PVX_113465	long chain polyunsaturated fatty acid elongation enzyme, putative	8
65	PVX_114095	cell division cycle protein 48 homologue, putative	8
66	PVX_114445	pyruvate kinase, putative	8
67	PVX_117925	elongation factor 2, putative	8
68	PVX_122930	histone H2B variant, putative	8
69	PVX_123845	polyadenylate-binding protein, putative	8
70	PVX_123935	haloacid dehalogenase-like hydrolase, putative	8
71	PVX_083555	hypothetical protein	7
72	PVX_090275	tryptophan-rich antigen (Pv-fam-a)	7
73	PVX_096985	variable surface protein Vir, putative	7
74	PVX_101515	tryptophan-rich antigen (Pv-fam-a)	7
75	PVX_112110	unspecified product	7
76	PVX_115460	Plasmodium exported protein, unknown function	7
77	PVX_003730	26S proteasome regulatory subunit RPN1, putative	7
78	PVX_080400	60S ribosomal protein L12, putative	7
79	PVX_080405	60S ribosomal protein L2, putative	7
80	PVX_080555	6-cysteine protein	7
81	PVX_080650	inositol-3-phosphate synthase, putative	7
82	PVX_081455	calcium-transporting ATPase, putative	7
83	PVX_083205	protein transport protein SEC61 subunit alpha, putative	7
84	PVX_083270	DNA/RNA-binding protein Alba 4, putative	7
85	PVX_084795	mitochondrial acidic protein MAM33, putative	7
86	PVX_085645	hypothetical protein, conserved	7
87	PVX_085735	60S ribosomal protein L10, putative	7
88	PVX_085830	actin II	7
89	PVX_086020	40S ribosomal protein S8e, putative	7
90	PVX_088205	vacuolar proton translocating ATPase subunit A, putative	7
91	PVX_089025	receptor for activated c kinase, putative	7
92	PVX_090950	40S ribosomal protein S4, putative	7
93	PVX_091790	hypothetical protein, conserved	7
94	PVX_092120	60S acidic ribosomal protein P0, putative	7
95	PVX_092310	heat shock protein 70, putative	7
96	PVX_092315	protein disulfide isomerase, putative	7
97	PVX_094375	40S ribosomal protein S20e, putative	7
98	PVX_097625	merozoite surface protein 8, putative	7
99	PVX_097845	hypothetical protein, conserved	7
100	PVX_098712	high molecular weight rhoptry protein 3, putative	7
101	PVX_099370	DNAJ-like molecular chaperone protein, putative	7
102	PVX_100735	ATP synthase subunit beta, mitochondrial, putative	7
103	PVX_123070	60S ribosomal protein L13, putative	7

Additional table 2. Sequence alignment data for unique proteins obtained from parasite pellets using pBLAST

Proteins	*P. falciparum*			*Homo sapiens*		
	Name	Identity	E value	Name	Identity	E value
PVX_094303	STP1 protein	39%	3e-95	-	-	-
PVX_003555	Plasmodium exported protein, unknown function	42%	9e-43	uncharacterized protein LOC107987394	33%	3e-04
PVX_003545	Plasmodium exported protein, unknown function	41%	6e-39	-	-	-
PVX_092990	tryptophan-rich protein	37%	1e-56	apolipoprotein B48 receptor, isoform CRA_a	44%	1.4
PVX_092995	tryptophan-rich protein	31%	2.00E-23	-	-	-
PVX_096975	PIR protein	37%	7.00E-60	-	-	-
PVX_112670	tryptophan-rich protein	48%	3.00E-108	-	-	-
PVX_001685	Plasmodium exported protein (PHIST), unknown function	53%	7.00E-51	-	-	-
PVX_094295	Plasmodium exported protein, unknown function	43%	1.00E-71	unnamed protein product	40%	8.7
PVX_096995	tryptophan-rich protein	65%	8.00E-164	-	-	-
PVX_118695	phist protein	37%	1.00E-31	KIAA0754 protein	20%	4.00E-15
PVX_121935	hypothetical protein PFMC_04220	21%	0.005	-	-	-
PVX_083555	Hypothetical protein POCGH01_12010100	55%	1.00E-22	-	-	-
PVX_096985	PIR protein	33%	1.00E-44	-	-	-
PVX_101515	tryptophan-rich protein	55%	5.00E-85	-	-	-
PVX_112110	Plasmodium exported protein (PHIST), unknown function	61%	9.00E-115	trophinin isoform 5	46%	0.004
PVX_115460	conserved Plasmodium protein, unknown function	42%	4.00E-05	-	-	-

Additional table 3. *P. vivax* proteins found in both plasma and parasite isolates

S. No	Gene ID	Product Description	Computed GO Components	Computed GO Processes
1	PVX_003545	Plasmodium exported protein, unknown function	membrane	-
2	PVX_003555	Plasmodium exported protein, unknown function	-	-
3	PVX_082845	elongation factor 1-gamma, putative	eukaryotic translation elongation factor 1 complex	translational elongation
4	PVX_083555	hypothetical protein	-	-
5	PVX_083560	Plasmodium exported protein, unknown function	-	-
6	PVX_087950	heat shock protein 86, putative	-	protein folding, response to heat, response to stress, response to unfolded protein
7	PVX_088960	protein disulfide isomerase, putative	endoplasmic reticulum, endoplasmic reticulum lumen	cell redox homeostasis, glycerol ether metabolic process, obsolete electron transport, protein folding
8	PVX_089425	heat shock 70 kDa protein, putative	-	response to heat, response to unfolded protein
9	PVX_089505	14-3-3 protein, putative	-	-
10	PVX_091515	GTP-binding nuclear protein RAN/TC4, putative	intracellular, membrane, nucleus	RNA export from nucleus, intracellular protein transport, nucleocytoplasmic transport, obsolete GTP catabolic process, protein transport, regulation of cell cycle, signal transduction, small GTPase mediated signal transduction
11	PVX_092065	spermidine synthase, putative	-	spermidine biosynthetic process
12	PVX_093680	Phist protein (Pf-fam-b)	apicoplast	-
13	PVX_094303	Pvstp1, putative	-	-
14	PVX_094635	tubulin beta chain, putative	cytoplasm, microtubule, protein-containing complex, tubulin complex	microtubule polymerization, microtubule-based movement, obsolete GTP catabolic process, protein polymerization
15	PVX_095000	heat shock protein 60, putative	cytoplasm, mitochondrion	cellular protein metabolic process, protein refolding, protein targeting to mitochondrion, response to unfolded protein
16	PVX_096070	early transcribed membrane protein (ETRAMP)	-	-

17	PVX_097583	skeleton-binding protein 1, putative	-	-
18	PVX_099315	heat shock protein 70, putative	-	-
19	PVX_099525	S-adenosylmethionine synthetase, putative	-	S-adenosylmethionine biosynthetic process, one-carbon metabolic process
20	PVX_101200	actin	actin cytoskeleton, actin filament	cytoskeleton organization
21	PVX_101400	osmiophilic body protein, putative	-	-
22	PVX_115450	hypothetical protein, conserved	-	-
23	PVX_117030	RNA helicase-1, putative	eukaryotic translation initiation factor 4F complex	regulation of translational initiation
24	PVX_117322	glyceraldehyde-3-phosphate dehydrogenase, putative	-	glucose metabolic process, oxidation-reduction process
25	PVX_117925	elongation factor 2, putative	-	translation, translational elongation
26	PVX_121935	Plasmodium exported protein, unknown function	-	-

Additional table 4A. GO classification of *P. vivax* proteins detected in plasma

Gene ID	Product Description	Gene Name or Symbol	Computed GO Components
PVX_001650	Pv-fam-d protein	null	-
PVX_003545	Plasmodium exported protein, unknown function	null	membrane
PVX_003555	Plasmodium exported protein, unknown function	null	-
PVX_081265	chromatin assembly factor 1 protein WD40 domain, putative	null	obsolete chromatin assembly complex
PVX_081832	Plasmodium exported protein, unknown function	null	-
PVX_082845	elongation factor 1-gamma, putative	EF1	eukaryotic translation elongation factor 1 complex
PVX_083555	hypothetical protein	null	-
PVX_083560	Plasmodium exported protein, unknown function	null	-
PVX_084940	voltage-dependent anion-selective channel protein, putative	null	mitochondrial outer membrane, mitochondrion
PVX_085275	60S ribosomal protein L5, putative	RPL5	cytosolic large ribosomal subunit, intracellular, mitochondrion, ribosome
PVX_087950	heat shock protein 86, putative	null	-
PVX_088960	protein disulfide isomerase, putative	null	endoplasmic reticulum, endoplasmic reticulum lumen
PVX_089425	heat shock 70 kDa protein, putative	null	-
PVX_089505	14-3-3 protein, putative	14-3-3I	-
PVX_090070	ran binding protein 1, putative	null	-
PVX_090160	40S ribosomal protein S19, putative	RPS19	intracellular, ribosome
PVX_090970	hypothetical protein, conserved	null	membrane
PVX_091105	endoplasmic reticulum-resident calcium binding protein, putative	ERC	endoplasmic reticulum
PVX_091515	GTP-binding nuclear protein RAN/TC4, putative	null	intracellular, membrane, nucleus
PVX_091640	phosphoglycerate mutase, putative	null	cytosol
PVX_092065	spermidine synthase, putative	null	-
PVX_093680	Phist protein (Pf-fam-b)	null	apicoplast
PVX_094303	Pvstp1, putative	null	-
PVX_094635	tubulin beta chain, putative	null	cytoplasm, microtubule, protein-containing complex, tubulin complex
PVX_095000	heat shock protein 60, putative	null	cytoplasm, mitochondrion
PVX_096070	early transcribed membrane protein (ETRAMP)	null	-
PVX_097583	skeleton-binding protein 1, putative	SBP1	-
PVX_099315	heat shock protein 70, putative	null	-
PVX_099525	S-adenosylmethionine synthetase, putative	null	-
PVX_099535	phosphoglycerate kinase, putative	null	-
PVX_101200	actin	null	actin cytoskeleton, actin filament
PVX_101400	osmiophilic body protein, putative	G377	-
PVX_115450	hypothetical protein, conserved	null	-
PVX_117030	RNA helicase-1, putative	null	eukaryotic translation initiation factor 4F complex
PVX_117322	glyceraldehyde-3-phosphate dehydrogenase, putative	GAPDH	-

PVX_117925	elongation factor 2, putative	EF2	-
PVX_121935	Plasmodium exported protein, unknown function	null	-
PVX_123745	endoplasmin, putative	GRP94	-

Additional table 4B. GO classification of *P. vivax* proteins detected in parasite isolates

Gene ID	Product Description	Gene Name or Symbol	Computed GO Components
PVX_003545	Plasmodium exported protein, unknown function	null	membrane
PVX_003555	Plasmodium exported protein, unknown function	null	-
PVX_082845	elongation factor 1-gamma, putative	null	eukaryotic translation elongation factor 1 complex
PVX_083555	hypothetical protein	null	-
PVX_083560	Plasmodium exported protein, unknown function	null	-
PVX_087950	heat shock protein 86, putative	null	-
PVX_088960	protein disulfide isomerase, putative	null	endoplasmic reticulum, endoplasmic reticulum lumen
PVX_089425	heat shock 70 kDa protein, putative	null	-
PVX_089505	14-3-3 protein, putative	14-3-3I	-
PVX_091515	GTP-binding nuclear protein RAN/TC4, putative	null	intracellular, membrane, nucleus
PVX_092065	spermidine synthase, putative	null	-
PVX_093680	Phist protein (Pf-fam-b)	null	apicoplast
PVX_094303	Pvstp1, putative	null	-
PVX_094635	tubulin beta chain, putative	null	cytoplasm, microtubule, protein-containing complex, tubulin complex
PVX_095000	heat shock protein 60, putative	null	cytoplasm, mitochondrion
PVX_096070	early transcribed membrane protein (ETRAMP)	null	-
PVX_097583	skeleton-binding protein 1, putative	SBP1	-
PVX_099315	heat shock protein 70, putative	null	-
PVX_099525	S-adenosylmethionine synthetase, putative	null	-
PVX_101200	actin	null	actin cytoskeleton, actin filament
PVX_101400	osmiophilic body protein, putative	G377	-
PVX_115450	hypothetical protein, conserved	null	-
PVX_117030	RNA helicase-1, putative	null	eukaryotic translation initiation factor 4F complex
PVX_117322	glyceraldehyde-3-phosphate dehydrogenase, putative	GAPDH	-
PVX_117925	elongation factor 2, putative	null	-
PVX_121935	Plasmodium exported protein, unknown function	null	-
PVX_001685	Phist protein (Pf-fam-b)	null	-
PVX_002650	40S ribosomal protein S26, putative	RPS26	intracellular, ribosome
PVX_003665	hexose transporter	null	integral component of membrane, membrane
PVX_003730	26S proteasome regulatory subunit RPN1, putative	RPN1	proteasome complex, proteasome regulatory particle
PVX_003905	6-cysteine protein	null	plasma membrane
PVX_080100	multidrug resistance protein 1, putative	MDR1	integral component of membrane
PVX_080400	60S ribosomal protein L12, putative	null	ribosome
PVX_080405	60S ribosomal protein L2, putative	RPL2	intracellular, large ribosomal subunit, ribosome

PVX_080440	40S ribosomal protein S11, putative	null	cytosolic small ribosomal subunit, ribosome
PVX_080555	6-cysteine protein	PSOP12	-
PVX_080650	inositol-3-phosphate synthase, putative	INO1	-
PVX_081455	calcium-transporting ATPase, putative	null	integral component of membrane
PVX_082840	60S ribosomal protein L6, putative	null	cytosolic large ribosomal subunit, intracellular, ribosome
PVX_083045	phosphoethanolamine N-methyltransferase	PMT	-
PVX_083205	protein transport protein SEC61 subunit alpha, putative	null	membrane
PVX_083240	6-cysteine protein	null	membrane
PVX_083270	DNA/RNA-binding protein Alba 4, putative	ALBA4	-
PVX_083515	protein disulfide-isomerase, putative	null	-
PVX_084795	mitochondrial acidic protein MAM33, putative	null	mitochondrial matrix
PVX_085645	hypothetical protein, conserved	null	membrane
PVX_085735	60S ribosomal protein L10, putative	null	cytosolic large ribosomal subunit, intracellular, ribosome
PVX_085830	actin II	null	actin cytoskeleton, actin filament
PVX_086020	40S ribosomal protein S8e, putative	null	cytosolic small ribosomal subunit, intracellular, mitochondrion, ribosome
PVX_088205	vacuolar proton translocating ATPase subunit A, putative	null	obsolete membrane fraction, proton-transporting V-type ATPase, V0 domain, proton-transporting two-sector ATPase complex, vacuolar proton-transporting V-type ATPase, V0 domain
PVX_089025	receptor for activated c kinase, putative	null	heterotrimeric G-protein complex
PVX_089635	phosphoglucomutase-2, putative	PGM2	-
PVX_090155	tubulin alpha chain, putative	null	microtubule, protein-containing complex, tubulin complex
PVX_090265	tryptophan-rich antigen (Pv-fam-a)	null	-
PVX_090275	tryptophan-rich antigen (Pv-fam-a)	null	-
PVX_090950	40S ribosomal protein S4, putative	null	intracellular, mitochondrion, ribosome, small ribosomal subunit
PVX_091470	heat shock protein 101, putative	HSP101	apicoplast
PVX_091700	circumsporozoite-related antigen, putative	EXP1	membrane
PVX_091790	hypothetical protein, conserved	null	membrane
PVX_092070	parasitophorous vacuolar protein 1, putative	PV1	-
PVX_092120	60S acidic ribosomal protein P0, putative	null	cytosolic large ribosomal subunit, intracellular, mitochondrion, ribosome
PVX_092310	heat shock protein 70, putative	null	-
PVX_092315	protein disulfide isomerase, putative	null	endoplasmic reticulum lumen
PVX_092990	tryptophan-rich antigen (Pv-fam-a)	null	-
PVX_092995	tryptophan-rich antigen (Pv-fam-a)	null	-
PVX_094295	Pv-fam-h protein	null	-
PVX_094375	40S ribosomal protein S20e, putative	null	cytosolic small ribosomal subunit, ribosome, small ribosomal subunit

PVX_094400	60S ribosomal protein L13a, putative	null	cytosolic large ribosomal subunit, large ribosomal subunit, ribosome
PVX_094535	RNA-binding protein, putative	null	-
PVX_095015	enolase, putative	null	phosphopyruvate hydratase complex
PVX_095080	40S ribosomal protein S3A, putative	null	cytosolic small ribosomal subunit, intracellular, ribosome
PVX_096265	40S ribosomal protein S5, putative	null	cytosolic small ribosomal subunit, small ribosomal subunit
PVX_096975	VIR protein	null	-
PVX_096985	variable surface protein Vir, putative	null	-
PVX_096995	tryptophan-rich antigen (Pv-fam-a)	null	-
PVX_097625	merozoite surface protein 8, putative	null	membrane
PVX_097845	hypothetical protein, conserved	null	-
PVX_097915	60S ribosomal protein L4, putative	RPL4	large ribosomal subunit, ribosome
PVX_098712	high molecular weight rhoptry protein 3, putative	RhopH3	-
PVX_099055	alkaline phosphatase, putative	null	-
PVX_099370	DNAJ-like molecular chaperone protein, putative	null	-
PVX_100735	ATP synthase subunit beta, mitochondrial, putative	null	integral component of membrane, mitochondrion, proton-transporting two-sector ATPase complex, proton-transporting two-sector ATPase complex, catalytic domain
PVX_100835	conserved protein, unknown function	null	membrane
PVX_101515	tryptophan-rich antigen (Pv-fam-a)	null	-
PVX_112110	unspecified product	null	-
PVX_112670	unspecified product	null	-
PVX_113465	long chain polyunsaturated fatty acid elongation enzyme, putative	null	integral component of membrane
PVX_114095	cell division cycle protein 48 homologue, putative	null	obsolete microsome
PVX_114315	hexokinase, putative	null	-
PVX_114445	pyruvate kinase, putative	null	-
PVX_115460	Plasmodium exported protein, unknown function	null	-
PVX_116915	exported protein 2, putative	EXP2	symbiont-containing vacuole membrane
PVX_117170	40S ribosomal protein S3, putative	null	cytosolic small ribosomal subunit, ribosome, small ribosomal subunit
PVX_117615	signal peptide peptidase, putative	SPP	apicoplast, integral component of membrane
PVX_117625	V-type H(+)-translocating pyrophosphatase, putative	VP1	membrane
PVX_118145	40S ribosomal protein S5, putative	null	cytosolic small ribosomal subunit, ribosome, small ribosomal subunit
PVX_118695	Pv-fam-d protein	null	-
PVX_121930	Plasmodium exported protein, unknown function	null	-
PVX_122930	histone H2B variant, putative	H2B.Z	nucleosome, nucleus
PVX_123070	60S ribosomal protein L13, putative	null	cytosolic large ribosomal subunit, intracellular, ribosome
PVX_123340	eukaryotic translation initiation factor 3 subunit 10, putative	null	eukaryotic translation initiation factor 3 complex

PVX_123845	polyadenylate-binding protein, putative	null	-
PVX_123935	haloacid dehalogenase-like hydrolase, putative	HAD3	-

Additional table 5. List of proteins and peptides selected for the MRM assay

S.no.	Peptides	Protein
1	LSEQELIQLLR	PVX_003545*
2	MPFDSPEQMLQFLK	
3	**TYLLLYTLSLVK**	
4	DVSNVAPLILPALPPLLMMFIGTQR	
5	LISSIFGFDEEDIDSR	PVX_083555
6	VTLESPDNAEIDMSS	
7	IESLDYLSLFK	
8	DNIFITNVLDQWIK	PVX_090265
9	GVSGLVPSSNSLQEITLR	
10	**LFGITSPEDTWLTDIEKPLVAK**	PVX_094303*
11	**ENLPDEIDVENLTK**	
12	**SALQSMLESFIK**	PVX_101520*
13	MYGVYDEQK	

*Proteins chosen for targeted proteomics assay. The peptides represented in bold showed consistent results among all the 8 plasma samples in the initial round of experiments

Additional figure 1. Representative peptides unique to *P. vivax* monitored using PRM assay

DNIFITNVLDQWIK

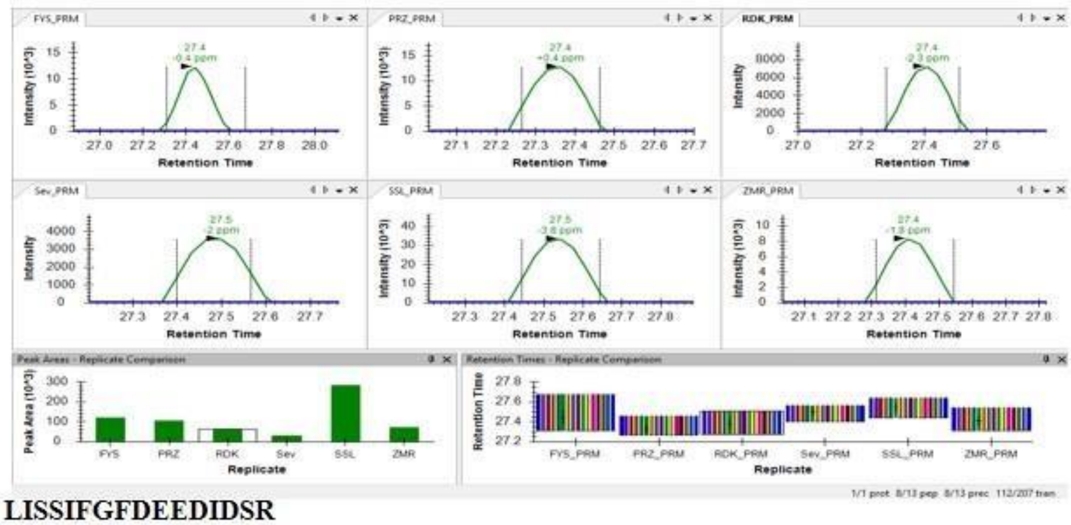

LISSIFGFDEEDIDSR

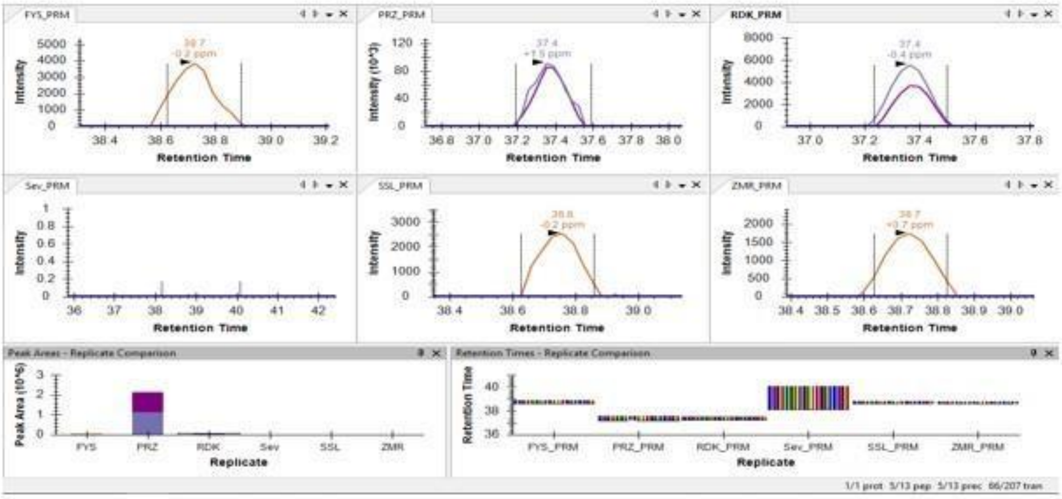

MPFDSPEQMLQFLK

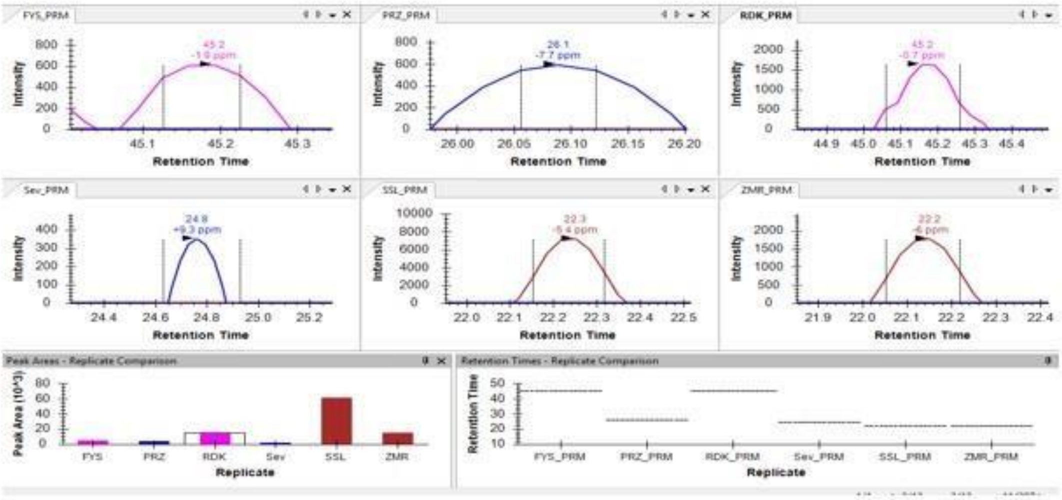

Additional figure 2. Sequence alignment of unique *P. vivax* proteins A) PVX_094303 B) PVX_003555 C) PVX_003545 D) PVX_121935 E) PVX_083555 and corresponding top *P. falciparum* hits using Clustal Omega. Certain regions of the protein sequences having highest dissimilarity between the two species are highlighted

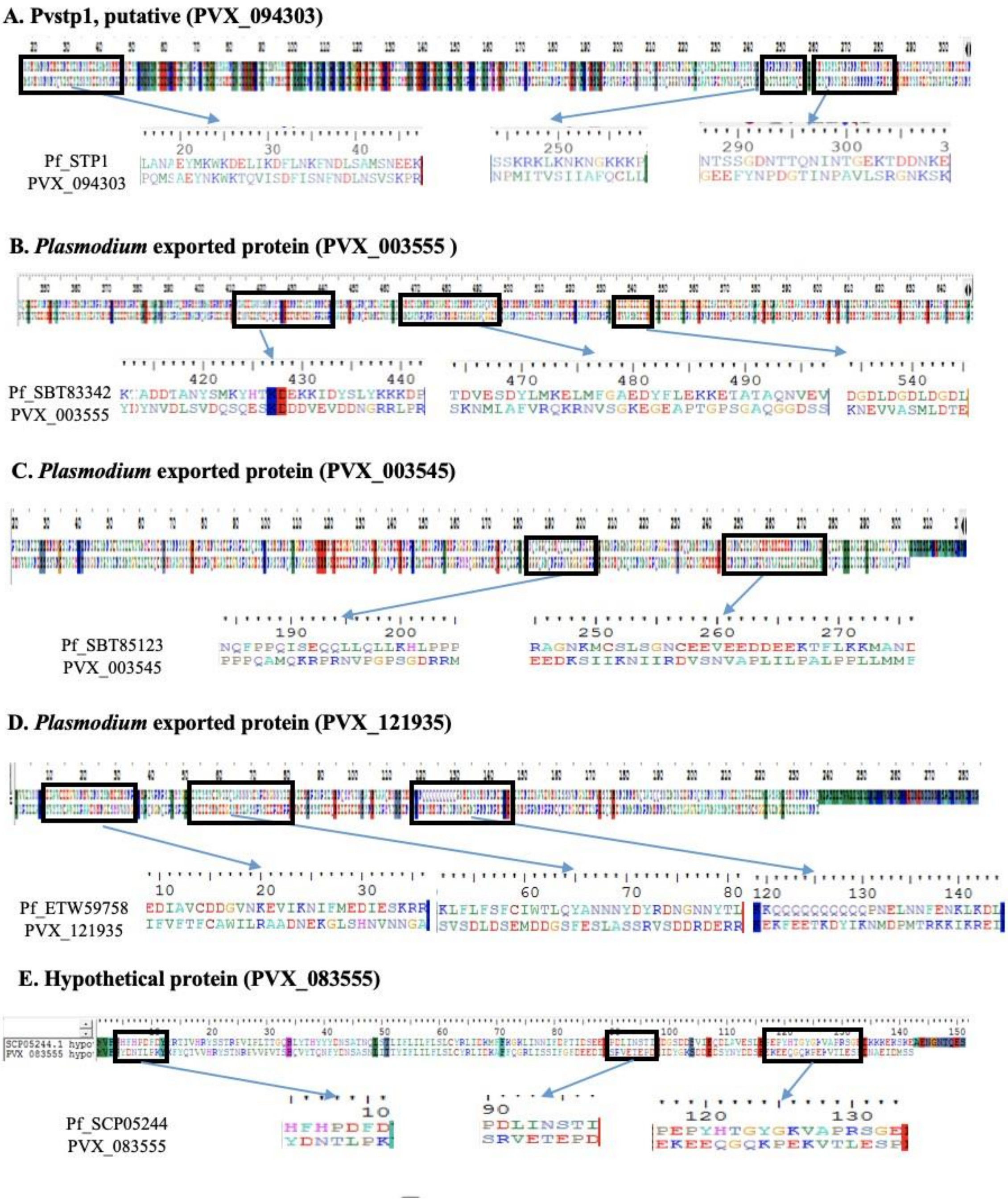

Additional figure 3. Standardization of peptide validation experiments using a targeted proteomics approach. The left panel shows a representative optimized MRM assay for sorted peptides. The top right panel shows the comparison of retention time in all the individual samples. The bottom left panel shows the comparison of Peak area in the plasma sample runs as Biological replicates. The experiment was performed for four peptides.

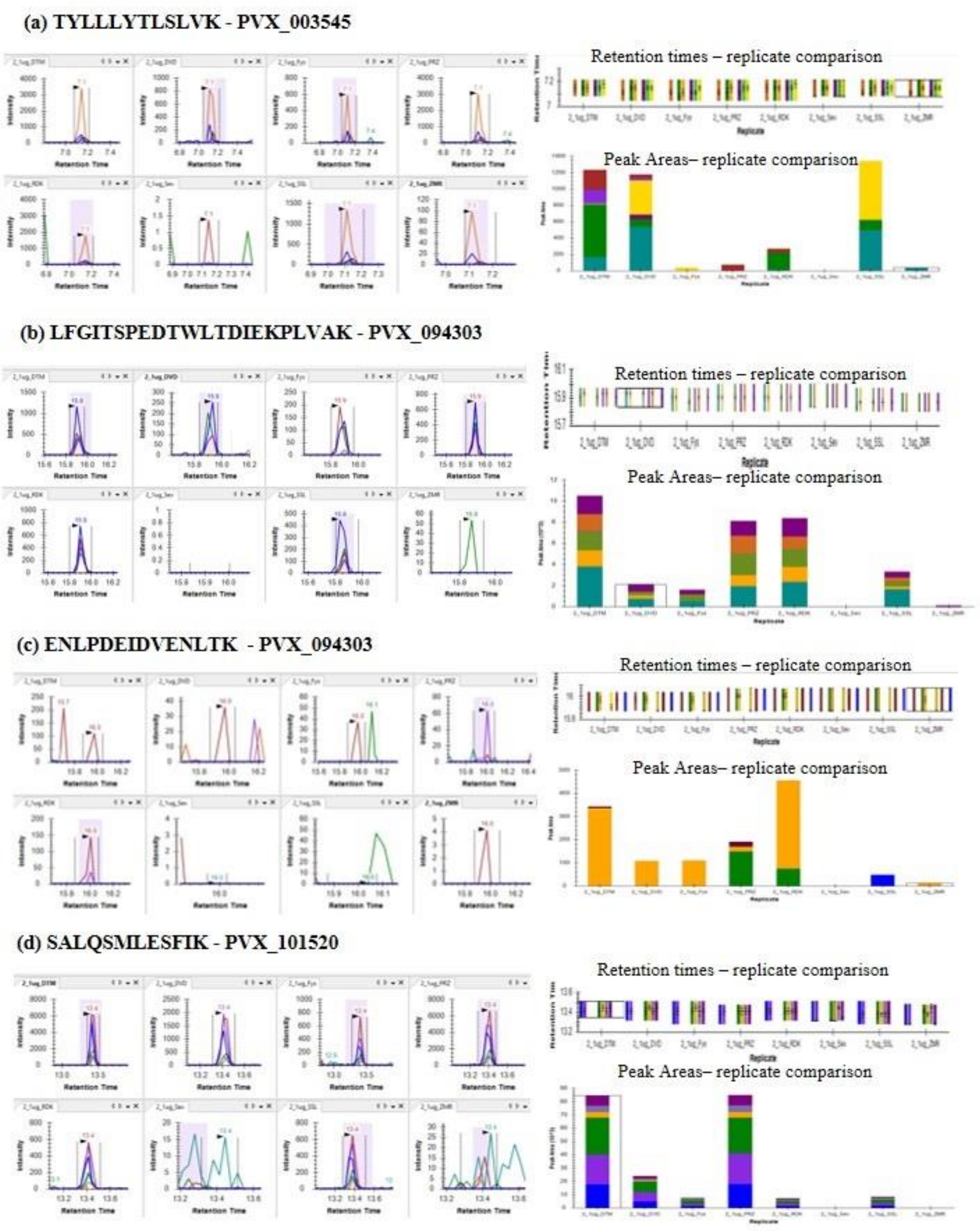

Supplement: Supplementary file 1 — Additional file 1: Table 1. P. vivax proteins in plasma samples and parasite isolates. Table 2. Sequence alignment data for unique proteins obtained from parasite pellets using pBLAST. Table 3. P. vivax proteins found in both plasma and parasite isolates. Table 4A. GO classification of P. vivax proteins detected in plasma. Table 4B. GO classification of P. vivax proteins detected in parasite isolates. Table 5. List of proteins and peptides selected for the MRM assay. Figure 1. Representative peptides unique to P. vivax monitored using PRM assay. Figure 2. Sequence alignment of unique P. vivax proteins. Figure 3. Standardization of peptide validation experiments using a targeted proteomics approach. [file 12879_2020_4885_MOESM1_ESM.pdf]
